# Is ragweed pollen allergenicity governed by environmental conditions during plant growth and flowering?

**DOI:** 10.1038/srep30438

**Published:** 2016-07-26

**Authors:** Alessandra Ghiani, Silvia Ciappetta, Rodolfo Gentili, Riccardo Asero, Sandra Citterio

**Affiliations:** 1 Dipartimento di Science Ambientali, Università di Milano-Bicocca, Piazza della Scienza 1, 20126 Milano, Italy; 2 Ambulatorio di Allergologia, Clinica San Carlo, Paderno Dugnano (MI), Italy

## Abstract

Pollen allergenicity is one of the main factors influencing the prevalence and/or severity of allergic diseases. However, how genotype and environment contribute to ragweed pollen allergenicity has still to be established. To throw some light on the factors governing allergenicity, in this work 180 ragweed plants from three Regions (Canada, France, Italy) were grown in both controlled (constant) and standard environmental conditions (seasonal changes in temperature, relative humidity and light). Pollen from single plants was characterized for its allergenic potency and for the underlying regulation mechanisms by studying the qualitative and quantitative variations of the main isoforms of the major ragweed allergen Amb a 1. Results showed a statistically higher variability in allergenicity of pollen from standard conditions than from controlled conditions growing plants. This variability was due to differences among single plants, regardless of their origin, and was not ascribed to differences in the expression and IgE reactivity of individual Amb a 1 isoforms but rather to quantitative differences involving all the studied isoforms. It suggests that the allergenic potency of ragweed pollen and thus the severity of ragweed pollinosis mainly depends on environmental conditions during plant growth and flowering, which regulate the total Amb a 1 content.

Common ragweed (*Ambrosia artemisiifolia* L.) is an annual weed, native from North America and accidentally introduced to Europe, probably with seed imports, during 19th century^1^. At the moment, in Europe, this alien plant is of great concern because it is one of the most prominent invasive species^1^,^2^,^3^. It has negative impacts on biodiversity, crop yields and human health causing respiratory allergies^4^,^5^,^6^,^7^. In this sense, *Ambrosia artemisiifolia* has been demonstrated to be one of the main allergenic species in Europe^8^. The major allergen of ragweed pollen is Amb a 1. More than 90% of ragweed-sensitized subjects react to Amb a 1 in skin prick tests and at least 90% of the allergenic activity in ragweed pollen can be attributed to this protein^9^. Amb a 1 belongs to the family of pectate lyase and shows many genetic isoforms (Amb a 1.1, Amb a 1.2, Amb a 1.3, Amb a 1.4, Amb a 1.5). Pollen allergenicity, defined as the pollen ability to elicit an IgE mediated response in atopic subjects and mainly due to the type and content of allergens, is widely recognized as a major determinant of health effects for sensitized patients and vary in pollen from single plants geographically and temporally^10^. Lee and Wang^11^, for example, demonstrated several-fold differences in allergenicity both among and within ragweed populations, although it was unclear whether these differences were genotypic or phenotypic. Several works demonstrated that pollution can influence the content of pollen allergens^12^,^13^,^14^,^15^. For instance Ghiani *et al*.^16^ reported an allergenicity increase of pollen from ragweed plants grown along polluted traffic roads. Climate change was also suggested to affect pollen allergenicity although, in this case, the research was primarily addressed to the study and prediction of the increasing temperature and CO_2_ concentration effects on pollen production and dispersion; only few studies were performed to define the role of climatic factors in altering pollen allergenic activity, which remains largely unexplored. For instance, it was shown that birch pollen content of the major allergen protein Bet v 1 increased at higher temperatures^17^ as well as ragweed Amb a 1 pollen content increased as function of rising atmospheric CO_2_ concentration^18^.

However, many other factors, both genetic and abiotic can influence allergen expression and it is still unknown if the antigen content is either function of environmental conditions or genetically determined. In addition pollen allergenicity is dependent not only on allergen amount but also on the type of allergens expressed; for example ragweed pollen allergenicity mainly depends on both the total content of Amb a 1 proteins and the presence of different Amb a 1 isoforms, whose single allergenic potential has not been completely elucidated yet.

In this work we studied the variation in Amb a 1 isoform expression and their IgE immunoreactivity among and within ragweed populations in order to determine the contribution of genetic and environmental factors to pollen allergenicity and to understand the underlying molecular mechanisms. Specifically, by growing ragweed plants both at standard conditions, where temperature (T), relative humidity (RH) and light (L) change during plant development, and in controlled conditions where environmental parameters (T, RH, L) are maintained constant throughout the whole plant life-cycle, we wanted to assess whether: (i) pollen allergenicity is genetically determined or is influenced by plant growth conditions; and (ii) if the differences among individuals reflect differences in the quantitative/qualitative expression of single isoforms.

## Results

### Total allergenicity of pollen from single plants

**Figure 1:**
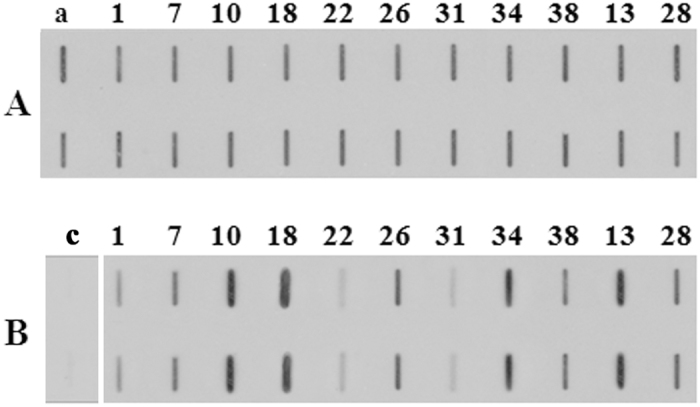
Representative slot blot membrane probed with a pool of selected patient sera showing the total allergenicity of pollen samples collected from plants grown in controlled (**A**) and standard (**B**) conditions. Each sample was loaded in two replicates. (a) Standard (protein extract from commercial pollen, Allergon); (c) negative control (serum pool from non-atopic subjects).

**Figure 2:**
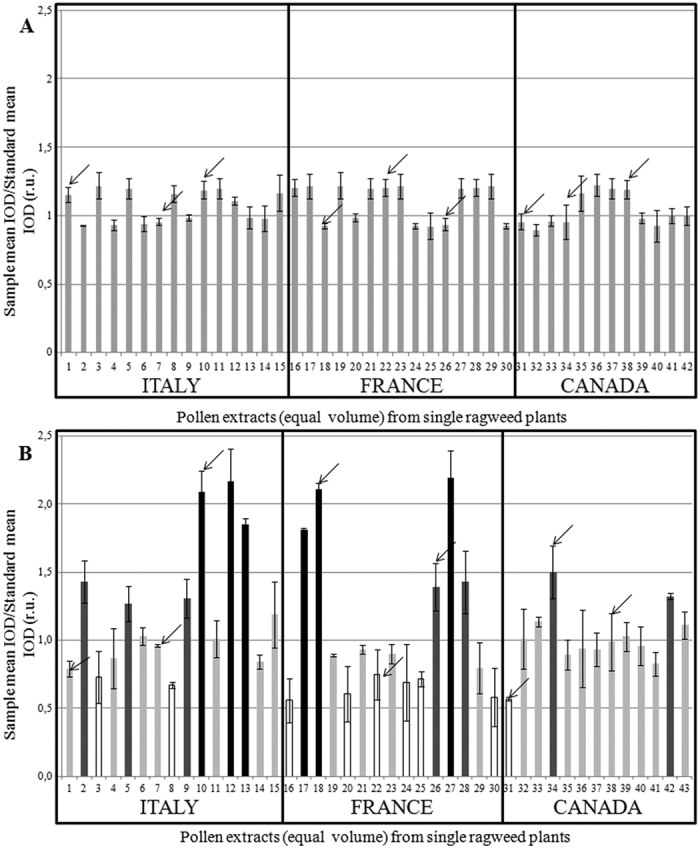
Assessment of total pollen allergenicity through image analysis. The integrated optical density (IOD) of immunoreactive spots with respect to the IOD of the standard (sample IOD/standard IOD) was measured. The results reported are the mean of five independent experiments. (**A**) plants grown in controlled conditions and (**B**) plants grown in standard conditions. The four reactivity clusters obtain by statistical analysis are shown with different colors; white bars: low reactivity (0.56 < IOD < 0.75); light grey bars: low-medium reactivity (0.78 < IOD < 1.19); dark grey bars: medium- high reactivity (01.27 < IOD < 1.50); black bars: high reactivity (1.81 < IOD < 2.19). Arrows indicate the 9 plants (randomly chosen within the low, medium and high IgE reactivity groups), that were used for 1D and 2D electrophoresis and immunoblotting analyses.

Slot blot technique was applied to assess the total pollen allergenicity of each of the plants grown in controlled or standard conditions. Identical and comparable volumes of soluble pollen extracts were bound on a nitrocellulose membrane and subjected to immunoreaction with a sera mix from selected ragweed allergic patients. Figure 1 shows representative membranes after immunodetection. Image analysis was applied to quantify immunochemical signals: the integrated optical density (IOD) of immunoreactive sposts with respect to the IOD of standard (sample IOD/standard IOD) was measured. At least 3 protein extracts from each plant were analyzed and the mean results of 5 independent experiments were calculated and statistically elaborated (Fig. 2). On average, the reactivity signal of pollen samples from plants grown in controlled conditions ranged from 0.89 to 1.22 with a variance value of 0.01 (Fig. 2A). No statistical difference was found among the mean IOD values calculated for Italian (1.07 ± 0.03), French (1.10 ± 0.04) and Canadian (1.03 ± 0.03) populations (P < 0.01). Pollen from plants grown in standard conditions showed reactivity signals ranging from 0.56 to 2.19 with a much higher variance (0.20; P < 0.001) than that of pollen samples from plant grown in controlled constant conditions (Fig. 2B). The mean IOD values of Italian (1.21 ± 0.12), French (1.09 ± 0.14), and Canadian (1.02 ± 0.06) populations were not statistically different for these plants too (P < 0.05). Independently from seed origin, the mean reactivity signals were statistically classified into four significantly different (P < 0.001) groups: (1) low: 0.56 < IOD < 0.75; (2) low-medium: 0.78 < IOD < 1.19; (3) medium-high: 01.27 < IOD < 1.50 and (4) high: 1.81 < IOD < 2.19.

### Expression and IgE-reactivity of Amb a 1 isoforms in pollen from single plants

**Figure 3:**
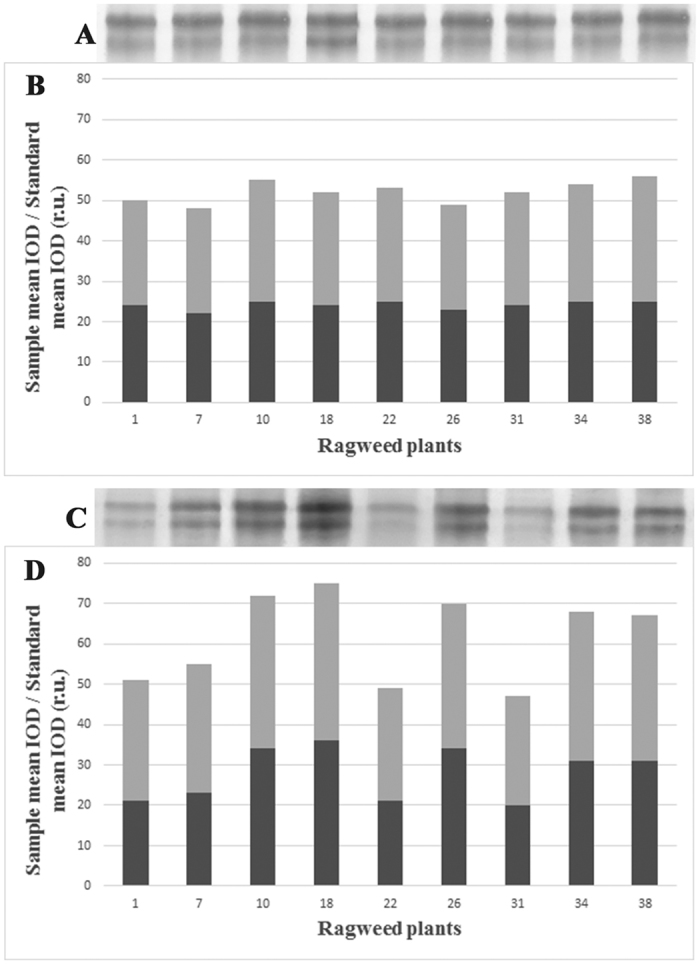
Representative SDS-PAGE gels showing Amb a 1 proteins from pollen of plants grown in controlled (**A**) and standard conditions (**C**) and related mean IOD calculated on 5 independent extracts of pollen from each plant (**B,D**, respectively). Light and dark grey: upper and lower band, respectively. Amb a 1 contents (IOD values) were related to the total content of proteins.

In order to understand if the high variability in total allergenicity found within plants grown in standard conditions was ascribed to different Amb a 1 amounts, 1D-SDS-PAGE and immunoblotting were carried out. Pollen from 9 plants, randomly chosen within the low, medium and high IgE reactivity groups resulting from the cluster statistical analysis of slot blot results, was analyzed. Additional 9 plants grown in controlled conditions were also considered as reference. Both equal volumes of extracts and equal amounts of proteins were loaded. Image analysis performed both on gels stained with Coomassie Blue and on the related immunoblot membranes, probed with the same sera mix used for slot blotting, confirmed the slot blot results and showed a significantly lower content of Amb 1 allergens (P < 0.05) in samples with lower reactivity (Fig. 3) indicating a direct relation between Amb a 1 content and pollen allergenicity (Pearson correlation = 0.98 P < 0,001 for standard group, 0.78 P < 0.05 for controlled group).

**Figure 4:**
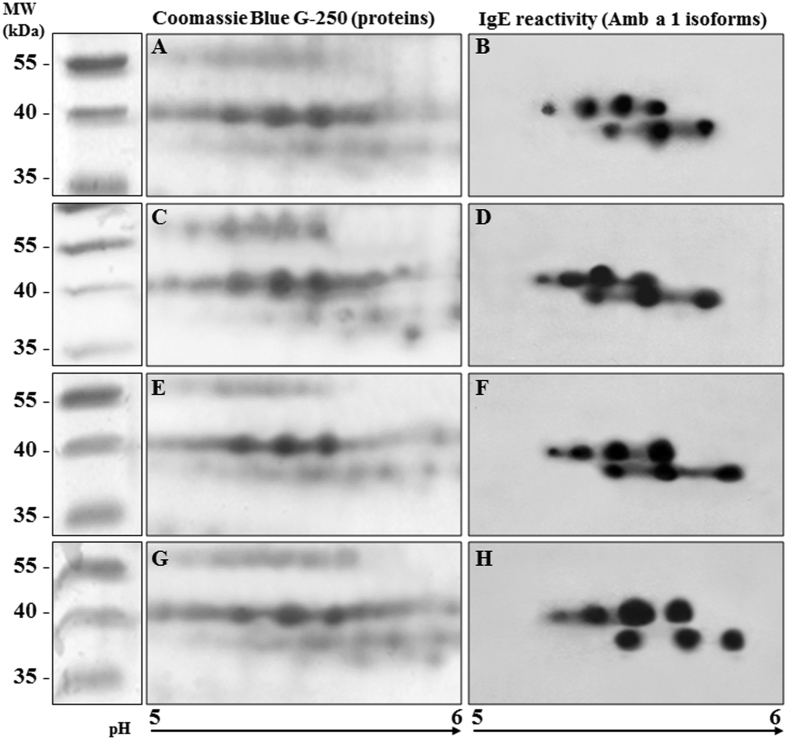
Expression and IgE-reactivity of Amb a 1 isoforms in pollen from plants grown in controlled (**A–D**) and standard conditions (**E–H**). A,C,E,G: examples of two dimensional gels stained with colloidal Coomassie Blue G-250; B,D,F,H: examples of two dimensional immunoblot membranes probed with a pool of selected patients’ sera.

The same pollen samples were in parallel analyzed with 2D-SDS-PAGE and immunoblotting to understand if a differential expression and/or reactivity of the single Amb a 1 isoforms contributed to the high variability in IgE reactivity of pollen from plants grown in standard conditions. In this case equal amounts of proteins were loaded on gels. Figure 4 shows representative membranes after immunodetection and the related gels stained with Coomassie Blue. No consistent variation in relative spot presence/intensity on both gels and membranes was observed by comparing them with our previously developed and published reference map^19^. It suggested that the great variability observed in allergenicity of pollen from plants grown in standard conditions was not ascribed to a different relative expression and IgE reactivity of the single isoforms considered.

On the whole electrophoresis and immunoblot analysis indicated that the variability in pollen allergenicity was mainly related to the pollen content of Amb a 1 allergen and not to a different relative expression and reactivity of its single principal isoforms.

## Discussion

The introduction and naturalization of *Ambrosia artemisiifolia* L. to Europe has impacted on human health, because this plant produces large quantities of highly allergenic pollen representing one of the main causes of pollinosis in many regions^8^. In Northern Italy, although present since the beginning of the XX century, common ragweed has become the second cause of respiratory allergy only in the last two decades^20^,^21^. Among the factors influencing pollinosis, the allergenic potency of pollen is an important element to be taken into account. In fact, in addition to all the anthropogenic and environmental changes leading to plant diffusion and to the release of greater amounts of pollen into the atmosphere, pollen allergenicity can also influence the prevalence and/or severity of allergic diseases. However, how genotype and environment contribute to determine ragweed pollen allergenicity is still to be established.

In this work we demonstrated that variations in ragweed pollen allergenicity are mostly due to seasonal climatic variations (T, RH, L) occurring during plant development and particularly during flowering time which is single-plant-specific. We found that pollen from plants grown in constant conditions show similar allergenic potency, whereas pollen from plants subjected to temperature, relative humidity and light seasonal changes show a high variability in allergenicity. Moreover, we found that also flowers of the same plant produce pollen with different allergenic potency only when they develop under variable conditions (data not shown).

Previous studies on ragweed, demonstrated that the content of its major allergen Amb a 1 vary in plants from site to site and even from year to year at the same site^11^,^22^. As for other allergenic plant species, the concentration of major pollen allergens may differ among plants as function of climatic conditions. For example, Ahlholm *et al*.^17^ indicated that birch pollen content of the major allergen protein Bet v 1 increased at higher temperatures and Saito and Teranishi^23^, by comparing the sugi major allergen Cry j 1 concentrations of four individuals of a clone growing at a low-altitude site and at a high-altitude site, found that the Cry j 1 concentration was higher in pollen collected at the low-altitude site. Specifically these authors speculated that differences in mean temperature of 1.1 and 1.5 °C, would cause changes in Bet v 1 and Cry j 1 allergen concentration, respectively. However Goto *et al*.^24^, by studying a greater number of sugi clones, reported that the Cry j 1 concentration was controlled primarily by genetic factors.

Our results indicate a principal control of ragweed pollen allergenicity mediated by environmental factors leading to phenotypic modifications not meiotically heritable. However, the low variability in allergenicity that we found among plants grown in constant controlled conditions suggest that genotypic heritable differences exist among plants and influence, although to a far lesser extent than environment, the final pollen allergenicity. It likely reflects the high gene flow existing among ragweed plants which determines a genetic variability that is very high among individual plants and very low among populations. Accordingly, in our experiments, allergenicity was single-plant-specific irrespective of the origin. Interestingly, also flowering time was single-plant-specific but the differences among plants were consistent and similar in constant and in standard conditions suggesting that this character, differently from allergenicity, is determined by genetic and/or epigenetic heritable factors. Moreover, in keeping with literature, reporting an increase in pollen Amb a 1 concentration induced by climatic changes and/or pollutants^18^,^25^,^26^, our experiment shows that environmental factors act through a molecular mechanism regulating the pollen content of all the considered Amb a 1 isoforms. A consistent preferential expression of specific Amb a 1 isoforms was, instead, not observed. This mechanism should involve an epigenetic control of pollen allergenicity as reported for many adaptation processes to environmental stresses. For instance El Kelish and collaborators^25^ observed an increase of the main Amb a 1 mRNAs in pollen of plants exposed to high concentrations of CO_2_ and under drought stress. We can speculate that the change in Amb a 1 pollen concentration could be a mechanism of adaptation acted by ragweed plants to favour their reproduction. Amb a 1 proteins belong, in fact, to the family of pectate lyases whose activity is implicated in pollen tube growth emergence by initiating the loosening and breaking of the pollen cell wall and also in pollen tube penetration in transmitting tissue^26^. Unfortunately, although epigenome modulation in response to the environment potentially provides a mechanism for organisms to adapt, both within and between generations, at present neither the extent to which this occurs, nor the molecular mechanisms involved are known^27^.

In summary, this study demonstrated that the allergenic potency of ragweed pollen, which directly affects the prevalence and/or severity of allergic diseases, is mainly governed by environmental factors. Specifically the experiments demonstrated that pollen allergenicity principally depends on the seasonally climatic changes occurring during plant development and particularly during flowering time. The research showed also that environment modulates the pollen potency by regulating the content of all the major Amb a 1 isoforms.

Our data provide a first step in identifying factors that are involved in the modulation of pollen allergenicity. Future experiments, aimed at investigating the direct relation between pollen allergenicity, single environmental parameters and epigenetic mechanisms, such as DNA methylation, are needed to gain further insight into the influence of environment changes on pollen allergenicity and into allergen physiological and ecological function.

## Methods

### Plant material

Seeds from 15 different ragweed populations (Table 1) were collected and used to obtain ragweed plantlets. To favor germination, collected seeds were subjected to a cold stratification (4 °C) for 30 days and then sown in a culture medium, consisting of 7% plant agar. Petri dishes were placed in a growth chamber (20 °C; 10 h dark/ 14 h light; 300 W m^−2^) and seeds were left to germinate. A total of 180 plantlets were transferred into 18 cm diameter pots containing universal soil. 90 plants out of 180 were grown in constant controlled conditions (25 °C; 10 h dark/ 14 h light; 300 W m^−2^) whereas the remaining 90 were transferred into a greenhouse and grown in standard conditions, where temperature (T), relative humidity (RH) and light (L) changed during season and then during plant development. The trends of T RH and total radiation during plant growth are reported in Supplementary Fig. S1.

Plants were monitored every day and pollen was collected by covering the male inflorescences with a plastic envelope (ARASYSTEM^®^, see Supplementary Fig. S2). Sampled pollen was stored in 2 ml eppendorf containing silica gel at room temperature.

### Immunochemical analysis

#### Patients and preparation of a sera mix

Sera from 12 adult subjects previously selected for their ability to specifically detect ragweed allergens^16^ were used to carry out all the immunochemical analyses. Specifically, adult subjects with a history of seasonal, summertime (mid August to end September) respiratory symptoms (rhino-conjunctivitis with or without asthma) who spontaneously presented at the allergy outpatient department of the Clinica San Carlo (Paderno Dugnano, Italy) asking for allergy evaluation, were considered as potential candidates for the inclusion in this study. All subjects underwent SPT with commercial extracts (Allergopharma, Reinbeck, Germany) of the main seasonal airborne allergens present in Italy, including ragweed, mugwort, grass, pellitory, plantain, birch, olive, and cypress and scored frankly positive on SPT with ragweed extract. All clinical investigations were carried out according to the principles of the Declaration of Helsinki; all patients gave their written informed consent to diagnostic procedures. The study was based on data stemming from routine clinical activity and on stored sera previously used to perform routine clinical investigations; the study has been approved by the Clinica San Carlo’s (Paderno Dugnano MI) Institutional Review Board. In order to avoid the interference of cross-reacting pan-allergens such as profilin, only patients sensitized to <3 pollens including ragweed were considered. Further, profilin hypersensitivity was ruled out by negative commercial profilin SPT (ALK-Abellò, Madrid Spain). The reactivity of 14 single sera was assessed by immunoblotting using a protein extract from commercial ragweed pollen (Allergon, Ängelholm, Sweden). Twelve sera were selected for their ability to specifically detect ragweed allergens and were pooled to carry out all immunochemical analyses (see Supplementary Fig. S3). The serum pool was aliquoted and stored at −20 °C until use.

#### Preparation of pollen protein extracts

Soluble protein extracts were prepared according to Aina *et al*.^12^ by suspending 0.1 g of pollen in 1 ml of bidistilled sterile water containing protease inhibitor (PMSF 1 mM). Samples were incubated on a rotating drum for 2 h at room temperature. The soluble fraction was isolated by means of two centrifugations at 13000 g for 10 min at 4 °C and then stored at −20 °C until use.

At least five independent extracts were prepared for each sample. Protein extracts were used for protein slot blot, 1D and 2D immunoblot analyses.

For Slot blot and 1D immunoblot analysis, samples were dissolved in SDS sample buffer [2% (w/v) SDS, 10% (v/v) glycerol, 1 mM DTT, 62.5 mM Tris-HCl, pH 6.8]. For 2D electrophoresis analysis, samples were purified with a clean-up kit (Bio-Rad Laboratories^®^) and dissolved in IEF rehydration buffer [7 M urea, 2 M thiourea, 2% (w/v) CHAPS, 20 mM Tris–HCl, pH 8.8, 20 mM DTT, 0.5% ampholyte mixture carrier, pH 3–10, 0.005% bromophenol blue]. Protein concentration was assayed according to Bradford^28^ using bovine serum albumin (BSA) as standard.

#### Protein Slot blot

Slot blot technique was applied to assess the whole allergenicity of pollen samples. The analysis was carried out according to Ghiani *et al*.^16^. Briefly, equal volumes of protein extracts (3 μl) were bound to nitrocellulose membrane and first stained with Ponceau S staining solution [0.1% (w/v) Ponceau S in 5% (v/v) acetic acid] to assess the amount of proteins loaded in each well. Membranes were then used to evaluate the immunoreactivity of the different pollen extracts to the sera mix from ragweed allergic patients. Protein extract from commercial pollen (Allergon) was used as standard to control staining variation when comparing measurements referring to different experiments. Negative controls were performed by omitting the sera mix and by using a pool of sera from non-atopic subjects. Image analysis was applied to quantify immunochemical signals: the integrated optical density (IOD) of immunoreactive spots with respect to the IOD of standard (sample IOD/standard IOD) was measured. The mean results of five independent experiments were calculated and statistically analyzed.

#### 1D and 2D immunoblotting

1D and 2D immunoblot analyses were performed to study the change in Amb a 1 isoform content and IgE reactivity among plants.

1D immunoblotting was carried out following the protocol reported by Aina *et al*.^12^. Briefly, equal volume of pollen extracts (15 μl/lane) or equal amounts of proteins (30 μg/lane) were separated by 14% SDS-polyacrylamide gels according to Laemmli^29^. Gels were either stained with colloidal Coomassie Blue G-250 (0.1% Coomassie Blue G250, 170 g/l ammonium sulphate, 34% methanol, 3% phosphoric acid) or transferred to nitrocellulose membrane. Membranes were blocked with 5% (w/v) non-fat dry milk powder in TBS-T [20 mM Tris, 150 mM NaCl and 0.05% (v/v) Tween 20, pH 7.5] for 1 h and then incubated for 16 h at 4 °C with a 1:10 dilution of the mixed sera from ragweed-allergic patients, and for control purpose, with sera from non-atopic adult individuals (1:10 dilution). Bound IgE were detected using an HRP-conjugated goat anti-human IgE antibody (1:15000 dilution; Sigma). Immunoreactive bands were visualized on an X-ray film (Kodak) using ECL Prime Western Blotting Detection (GE Healthcare^®^).

2D immunoblotting was performed according to Asero *et al*.^19^. Isoelectrofocusing (IEF) was carried out on 7 cm long immobilized pH gradient (IPG) strips (Bio-Rad^®^), providing a linear pH 4–7 gradient. Strips were rehydrated in 200 μl of rehydration buffer (7 M urea, 2 M thiourea, 2% (w/v) CHAPS, 20 mM DTT, 0.5% ampholyte mixture carrier, pH 3–10, 0.005% bromophenol blue) containing 100 μg of protein sample. Passive rehydration (up to 10 hours) and IEF were performed at 20 °C using a Protean IEF-Cell (Bio-Rad Laboratories^®^). After the first dimension separation, the IPG strips were equilibrated for 15 min against 6 M urea, 30% glycerol, 2% SDS, 0.375 M Tris–HCl pH 8.8, 2% DTT, in order to resolubilize proteins and reduce disulfur bonds. The –SH groups were then blocked by substituting the DTT with 2.5% iodoacetamide in the equilibration buffer for 15 min. After equilibration, strips were placed on the top of vertical 10 × 9 cm × 1.5 mm polyacrylamide gels (14% v/v). An agarose solution (0.5% low melting agarose in running buffer) was loaded to the top of the gel to lock strips, and electrophoresis was performed at 4 °C in a Laemmli running buffer (25 mM Tris–HCl pH 8.3, 192 mM glycine, 0.1% SDS). Gels were run in the electrophoresis chamber (Mini-Protean electrophoresis system, Bio-Rad Laboratories^®^) in parallel and used for protein revealing or immunoblotting experiments. For proteins detection, gels were stained with colloidal Coomassie Blue G250 (0,1% Coomassie Blue G250, 170 g/l ammonium sulfate, 34% methanol, 3% phosphoric acid). For immunodetection experiments, gels were electroblotted (100 mA, overnight at 4_C) onto nitrocellulose membranes (0.45 mm, Bio-Rad Laboratories) by a Trans-Blot cell apparatus (Bio-Rad Laboratories) that contained transfer buffer (25-mmol/L Tris, 192-mmol/L glycine, and 20% [vol/vol]methanol, pH 8.3). Nitrocellulose filter saturation and sera-mix reaction were performed as reported above for 1D immunoblotting. At least 3 independent samples for each selected plant were analyzed. Amb a 1 isoform identification was made by image analysis, comparing the 2D maps with the ragweed reference map previously published by Asero *et al*.^19^. Before the comparison, a few gels were analyzed by LC-MS/MS to confirm the previous results. As previously found, independently of where plants were grown (standard or constant condition), Amb a 1.01 and Amb a 1.03 were the most expressed and IgE reactive isoform in pollen, respectively; Amb a 1.02 and Amb a 1.04 were instead the lower expressed isoforms.

### Statistics

Data were analyzed by R program for Windows. ANOVA and Turkey’s test, for multiple sample comparison, were applied when normality and homogeneity of variance were satisfied. Data, which did not conform to the assumptions, were transformed into logarithms.

## Additional Information

**How to cite this article**: Ghiani, A. *et al*. Is ragweed pollen allergenicity governed by environmental conditions during plant growth and flowering? *Sci. Rep.* **6**, 30438; doi: 10.1038/srep30438 (2016).

## Supplementary Material

Supplementary Information

## Figures and Tables

**Table 1:** Location of ragweed populations selected as source of seeds.

Pop	Locality	State	N	E
MM	Magenta	Italy	45°27′15″	8°53′46″
L	Lodi	Italy	45°18′52″	9°31′05″
BR	Brescia	Italy	45°29′23″	10°11′47″
G	Greco	Italy	45°30′26″	9°12′39″
P	Pavia	Italy	45°11′43″	9°10′05″
26P18	Allex	France	45°44′52″	4°55′04″
39P04	Saint Germain les Arlay	France	46°45′56″	5°34′25″
01P01	Ambronay	France	45°59′35″	5°19′37″
26P21	Livron sur Drôme	France	44°46′02″	4°50′45″
26P19	Grane	France	44°44′58″	4°52′56″
LOT 18	Mirabel	Canada	45°39′45″	73°00′10″
LOT 878	Ste Clotilde de Chateauguay	Canada	45°11′24″	73°38′59″
LOT 6	L’Acadie	Canada	45°18′52″	73°21′19″
LOT 800	Ste Clotilde de Chateauguay	Canada	45°09′49″	73°40′17″
LOT 990	Ste Clotilde de Chateauguay	Canada	45°09′17″	73°41′02″
