# Supplementary material for: Is ragweed pollen allergenicity governed by environmental conditions during plant growth and flowering?
Source: Sci Rep. 2016 Jul 26;6:30438. doi: 10.1038/srep30438 (PMC4960655; doi:10.1038/srep30438)
Supplement: Supplementary Information [file srep30438-s1.pdf]

Supplementary Information

Is ragweed pollen allergenicity governed by environmental conditions during plant growth and flowering?

Alessandra Ghiani[1a], Silvia Ciappetta[1a], Rodolfo Gentili[1], Riccardo Asero[2], S. Citterio[1*]

[1] Dipartimento di Science Ambientali, Università di Milano-Bicocca, Piazza della Scienza 1, 20126 Milano, Italy.

[2] Ambulatorio di Allergologia, Clinica San Carlo, Paderno Dugnano (MI), Italy.

[a] Alessandra Ghiani and Silvia Ciappetta contributed equally to this work

***Corresponding author:**

Sandra Citterio, Dipartimento di Science Ambientali, Università di Milano-Bicocca, Piazza della Scienza 1, 20126 Milano, Italy.

Tel.: +39 02 64482934

Fax: +39 02 64482996

E-mail: sandra.citterio@unimib.it

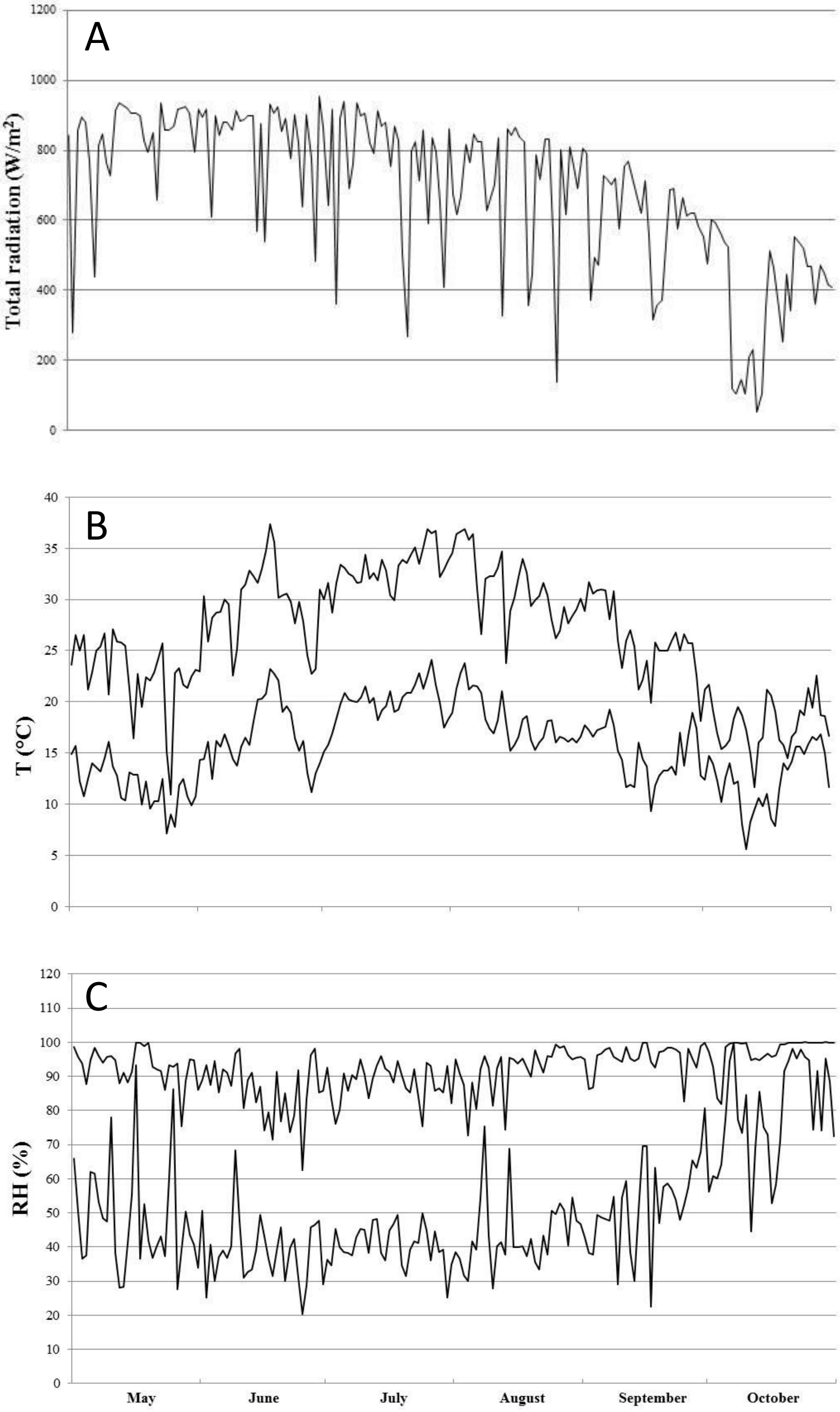

Figure S1. Trends of total radiation (W m^{-2}, daily maximum value), relative humidity (RH) and temperature (T) during plant growth in standard condition. The percentage of UVA radiation ranged

from 2 to 4% and the percentage of UVB was less than 0.1%. The two lines within B and C graphics represent the minimum (lower line) and maximum (upper line) daily value.

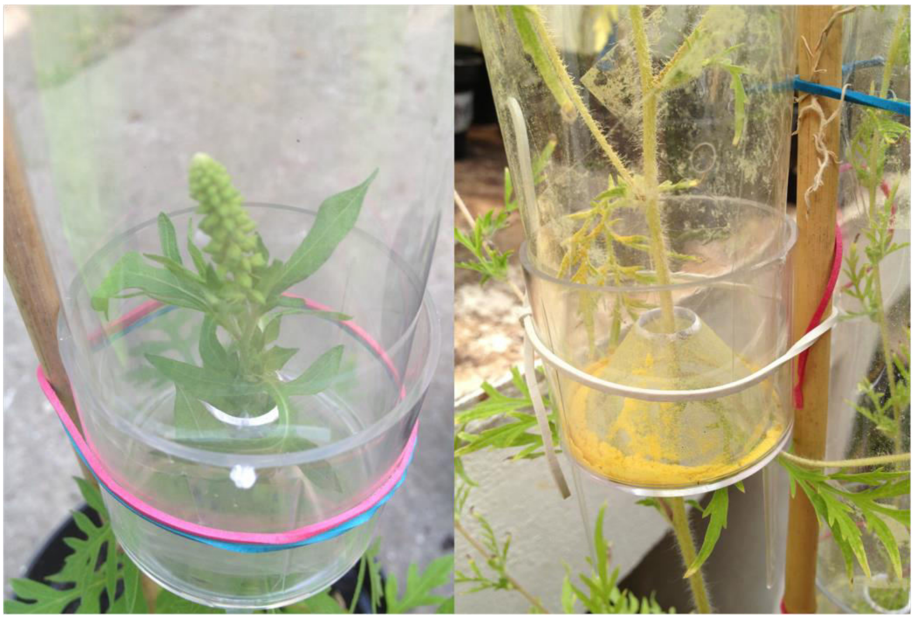

Figure S2. Male inflorescences covered with a specific pollen collection system (modified ARASYSTEM®).

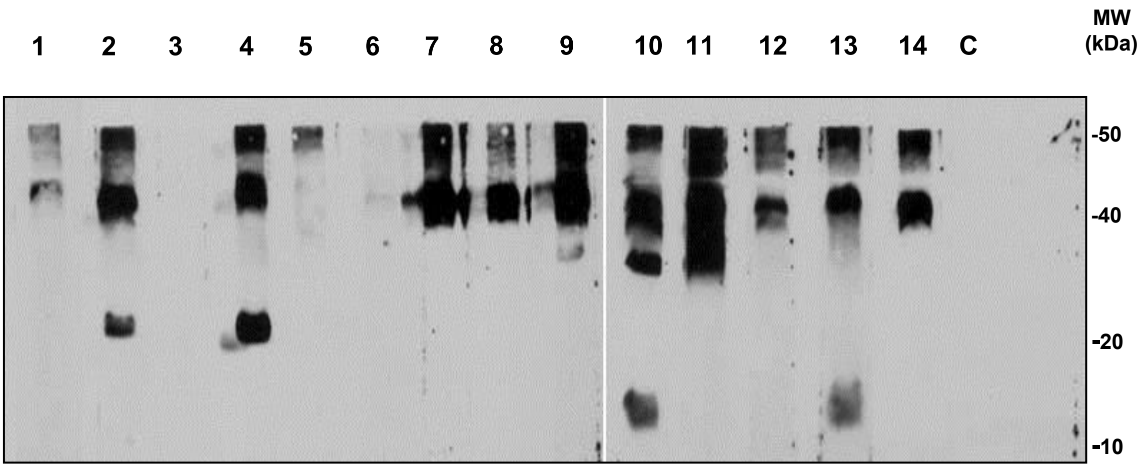

Figure S3. Immunoblot membrane showing the Ig-E reactivity of 14 single patients' sera to proteins extracted from commercial ragweed pollen (Allergon, Ängelholm, Sweden). For immunochemical analysis, 5 ml of sera number 1, 5, 7, 8, 9, 10, 11, 12, 13, 14 were combined and mixed with a double volume (10 ml) of sera number 2, 4, 10, 13. A total volume of 90 ml of serum pool was obtained; it was aliquoted and stored at -20°C until use.

1-14: serum identification number; C: IgE reactivity of a serum pool from three non-atopic subjects (control).
